# Diclofenac-Induced Immune Hemolytic Anemia: A Case Report and Review of Literature

**DOI:** 10.7759/cureus.12903

**Published:** 2021-01-25

**Authors:** Alexandra Esteves, Francisco Teixeira da Silva, José Carvalho, Ana Carvoeiro, Paula Felgueiras

**Affiliations:** 1 Internal Medicine, Unidade Local de Saúde do Alto Minho, Viana do Castelo, PRT

**Keywords:** diclofenac, hemolysis, immune hemolytic anemia, anemia, drug-induced immune hemolytic anemia

## Abstract

Non-steroidal anti-inflammatory drugs are widely used for pain management. Most frequently, adverse reactions affect the gastrointestinal tract and hematological side effects usually relate to the gastrointestinal manifestations. Drug-induced immune hemolytic anemia is a rare and frequently underdiagnosed complication that is associated with poor outcomes including organ failure and even death.

A 76-year-old female patient was treated with intramuscular diclofenac, thiocolchicoside, and diazepam for low back pain. Five days following diclofenac exposure, the patient was admitted to the Emergency Department with complaints of asthenia, nausea, vomiting, and diarrhea. Hemolysis and a positive direct antiglobulin test were detected on laboratory testing. Further causes of hemolytic anemia were excluded and a diagnosis of diclofenac-induced immune hemolytic anemia was established. Glucocorticoid therapy initiated on admission and drug eviction led to complete recovery. Long-term follow-up showed no recurrence of anemia.

Here, we present the unusual case of a successful recovery of a 76-year-old patient with diclofenac-induced immune hemolytic anemia, a rare but immediate life-threatening condition of a frequently used drug in clinical practice.

## Introduction

Autoimmune hemolytic anemia (AIHA) ensues when the host’s immune system acts against its own red cell antigens and has an estimated prevalence of approximately 1 in 100,000 individuals [1]. Approximately 50% of the cases refer to primary or idiopathic AIHA, where an associated disorder is not found [2]. Secondary causes of AIHA depend on the studied population. Current series estimate that half are associated with hematological malignancy, a third with infection, a sixth with collagen vascular disorders, and a tenth with drug-induced immune hemolytic anemia (DIIHA), the latter reaching an estimated incidence of one per million per year [1,3].

Diclofenac is one of the non-steroidal anti-inflammatory drugs (NSAIDs) most used for the treatment of rheumatoid arthritis and osteoarthritis [4]. Though generally well tolerated, over 400 adverse reactions have been documented. Most frequently, adverse reactions affect the gastrointestinal tract, the skin, and the central nervous system [5]. Direct hematological side effects such as leukopenia, thrombocytopenia, and aplastic anemia have been described only in limited cases [6-9].

We present the case of a 76-year-old patient with diclofenac DIIHA and a summary of the pathophysiology and therapeutic options.

## Case presentation

A 76-year-old woman presented to the Emergency Department (ED) with recent onset of fatigue. She had a previous medical history of essential arterial hypertension, dyslipidemia, and spinal osteoarthritis with sporadic episodes of lumbosciatic pain. Regular medications initiated several years prior included perindopril 8 mg and rosuvastatin 10 mg. No allergies, alcohol, tobacco, toxins, or animal exposures were known, and she had no other relevant personal or familiar history.

Three weeks before admission the patient had an exacerbation of right lumbosciatic pain. This episode was similar to the previous ones, for which she usually was prescribed oral NSAIDs, acetaminophen, general physical therapy, massages, and rest with complete recovery. However, this time the pain was refractory to general measures, and eight days before admission, she was prescribed a combination of a daily intramuscular administration of 4 mg thiocolchicoside, 75 mg diclofenac, and 5 mg diazepam for a total of six days.

On the fifth day of treatment, she developed generalized malaise, fatigue, nausea, postprandial vomiting, and diarrhea with up to six soft, brownish dejections per day.

Despite resolution of nausea, vomiting, and diarrhea, she experienced progressive worsening of fatigue and was admitted to the ED. Detailed medical history was negative for other symptoms, namely, fever, coluria, acholia, melena, and other evident blood losses, either on admission or in the past.

Physical examination revealed jaundice and pallor of the skin and mucous membranes. No epistaxis, gingivorrhagia, adenopathies, ecchymosis, or other skin lesions were found. Blood pressure was 137/62 mmHg and heart rate was 96 beats per minute, respiratory rate was 16 beats per minute, and peripheral oxygenation saturation was 96%. No fever or other abnormalities were noted.

Blood examination showed normocytic normochromic anemia (hemoglobin: 7.9 g/dL, reference median globular volume: 88 fL), reticulocytosis (9.2%), leucocytosis (white blood cells: 13.8 × 10 9/L), hyperbilirubinemia at the expense of unconjugated bilirubin (total bilirubin: 4.09 mg/dL, conjugated bilirubin: 0.97 mg/dL), elevated lactic dehydrogenase (805 UI/L), and sedimentation rate (VS: 76 mm). Serum iron concentration, ferritin, total iron-binding capacity, folic acid, or vitamin B12 showed no significant changes and haptoglobin levels were undetectable. Peripheral blood smear revealed exuberant erythrocyte rouleaux and spherocytes. The direct antiglobulin test (DAT) was positive for immunoglobulin G (IgG). Chest radiography, abdominal ultrasound, and electrocardiogram performed in the ED were normal.

The hypothesis of AIHA was considered in the ED. The patient was started on 60 mg of prednisolone per day (1 mg/kg/day) and was admitted to the medical ward.

The hypothesis of secondary AIHA to an infectious or a connective tissue disease was ruled out. Gastrointestinal complaints were compatible with side effects of NSAIDs, viral serologies, blood cultures, rheumatoid factor, antinuclear antibody, antineutrophil cytoplasmic antibodies, and cyclic citrullinated peptide antibody were negative, and complement (both C3 and C4 studies) was normal. Lymphoproliferative disease was excluded due to the absence of adenopathies both on physical examination and in imaging studies, as well as normal immunoglobulin levels (IgG, IgM, and IgA) and a normal serum protein electrophoresis. Inflammatory bowel disease and digestive tract tumors were also excluded by endoscopic observation and anatomopathological analysis of biopsies made in macroscopically normal mucosa.

During hospitalization, glucocorticoid therapy was maintained, and folic acid supplementation of 5 mg per day was started. The patient had clinical improvement with resolution of asthenia and jaundice, as well as progressive normalization of the hemoglobin and bilirubin values. On discharge, 13 days after admission, anemia was mild (hemoglobin: 11 g/dL) and no signs of hemolysis were noted. Table 1 shows the laboratory data during hospitalization.

**Table 1:** Laboratory data during hospitalization. ALP: alkaline phosphatase; ALT: alanine aminotransferase; ANA: antinuclear antibodies; ANCA: antineutrophil cytoplasmic antibody; anti-CCP: anti-cyclic citrullinated peptides; AST: aspartate aminotransferase; GGT: gamma-glutamyl transferase; HBV: hepatitis B virus; HCV: hepatitis C virus; HIV: human immunodeficiency virus; Ig: immunoglobulin; LDH: lactate dehydrogenase, MCV: mean corpuscular volume, TIBC: total iron binding capacity

		Results					
Variable	Reference range (adults)	Day 1	Day 2	Day 5	Day 7	Day 9	Day 13
Hemoglobin (g/dL)	11.8-15.8	7.9	7.8	8.2	9.1	10.3	11
Hematocrit (%)	36.0-46.0	21.9	22.1	24.4	26.7	30.6	33
Red blood cells (10^6^/µL)	4.2-5.4	2.47	2.42	2.48	2.63	2.97	3.14
MCV (fL)	80.4-96.4	88.7	91.3	98.4	101.5	103	105.1
White cell count (10^3^/µL)	4.0-10.0	13.88	12.49	18.09	15.62	11.43	11.82
Differential cell count (10^3^/L)							
Neutrophils	1.5-8.0	9.1	8.3	11.448	9.4	5.9	7.2
Lymphocytes	0.8-4.0	3.3	2.7	5.5	5.2	4.7	3.8
Platelet count (10^3^/µL)	150-400	353	353	381	377	392	362
Reticulocytes (%)	0.5-1.5	9.22					
Sedimentation rate (mm)	4-10	103					9
C-reactive protein (mg/dL)	<0.51	3.23	2.67	0.69	0.41	0.23	0.14
Urea (mg/dL)	17.0-43.0	40	31	39	35	29	33
Creatinine (mg/dL)	0.6-1.0	0.84	0.78	0.8	0.78	0.84	0.79
Sodium (mmol/L)	136-145	140	141	138	143	141	142
Potassium (mmol/L)	3.5-5.1	4.2	4.2	3	3.7	4	3.9
Calcium (mg/dL)	8.6-10.3		8.7				
Uric acid (mg/dL)	2.6-6		7				
Bilirubin (mg/dL)							
Total	0.3-1.2	4.09	4.01	2.16	2.29	2.27	1.28
Direct	<0.5	0.07	0.82	0.71	0.75	0.75	0.46
LDH (UI/L)	125-220	805	632	467	436	388	309
ALP (UI/L)	30-120	152	135	96	93	90	76
GGT (UI/L)	<38	83	70	55	56	54	43
ALT (UI/L)	7-45	44		19	23	30	31
AST (UI/L)	8-35	30	22	15	21	21	23
Iron (µ/dL)	70-180	124					
TIBC (µ/dL)	250-245	300					
Folic acid (ng/mL)	2.34-17.56	5.8					
Vitamin B12 (pg/mL)	187-883	414					
Haptoglobin (mg/dL)	35-250	<8					
Direct antiglobulin test		Positive for IgG					
Protein electrophoresis			No monoclonal spikes				
Total proteins (g/dL)	6.4-8.2		6.1				
Albumin (g/dL)	3.5-5.2		3.6				
Immunoglobulins							
IgA (mg/dL)	60-400		192				
IgG (mg/dL)	70-1,600		1,008				
IgM (mg/dL)	40-230		131				
ANA			Negative				
ANCA			Negative				
Rheumatoid factor				Negative			
anti-CCP				Negative			
Serology							
HIV			Negative				
HBV			Negative				
HCV			Negative				

Given the acute clinical context after diclofenac intake and absence of evidence for other secondary etiologies, the diagnosis of acquired hemolytic anemia secondary to NSAIDs was made. Follow-up three years later found the patient asymptomatic with no recurrence of anemia or hemolysis.

## Discussion

Drugs are generally small molecules unable to elicit an immune response, however, they can function as haptens, bind to larger proteins, become immunogenic, and lead to antibody production. These antibodies can be further classified as drug-induced, drug-dependent, and drug-independent antibodies. Drug-induced antibodies can bind to red blood cells (RBCs) and lead to hemolysis by non-immunological modification of erythrocyte membranes known as adsorption of non-immunological proteins. Drug-dependent antibodies bind to RBCs only in the presence of the drug or its metabolites, causing abrupt complement-mediated intravascular hemolysis. In vitro tests searching for lysis, DAT, or indirect antiglobulin test can be performed to confirm the diagnosis using the offending drug or its metabolites and the patient’s plasma or serum. Drug-independent antibodies can react with RBCs even in the absence of the drug and, therefore, are indistinguishable from autoantibodies mediating warm autoimmune hemolytic anemia (WAIHA) [1,10,11].

WAIHA is named after warm agglutinins, usually IgG autoantibodies, that bind to antigens on erythrocytes at a temperature of 37°C, leading to RBC destruction and a chronic or relapsing disease with an almost pathognomonic positive DAT [2].

Patients are treated with glucocorticoids 1 to 2 mg/kg of body weight/day of prednisone administered orally or an equivalent dose of methylprednisolone administered intravenously. Though most patients recover to an hemoglobin level above 10 g/dL within two to three weeks of treatment, relapse after treatment discontinuation is common, with retrospective case studies suggesting long-term remission rates as low as 20% to 30% [2].

DIIHA is identified by clinical evidence of hemolysis associated with drug therapy. Although many drugs have anedoctally reported isolated cases of DIIHA, over 130 drugs have reasonable evidence that supports an immune etiology [12]. It has been speculated that DIIHA is far more common than previously estimated, as most reports usually refer to more severe cases, either presenting with shock, multiorgan damage, or renal failure, and most mild cases being unreported [6].

Drugs most frequently associated with DIIHA are second and third generation of cephalosporins, diclofenac, oxaliplatin, and fludarabine. In European cohorts where diclofenac is the most frequently used NSAID, case reviews reported diclofenac as the most frequently implicated drug, with a fatal outcome being estimated in 15% to 21% of the reported cases [6,12-16]. Diclofenac-induced immune hemolytic anemia has been demonstrated in few cases, where the exposure to the drug or its metabolites lead to developing both drug-independent IgG autoantibodies and antibodies that reacted with diclofenac and its metabolites [8].

Signs and symptoms of DIIHA, as any other hemolytic anemia, are proportional to the degree and time of onset and can present withing hours to weeks of drug exposure. These include fatigue, dyspnea, and thoracic or abdominal pain. Late symptoms are usually due to complications of decompensated hemolysis, usually shock or renal failure. Laboratory abnormalities include low hemoglobin and haptoglobin levels, elevated lactate dehydrogenase and indirect bilirubin levels, reticulocytosis, and a positive DAT [1,10].

The DAT is positive in all the described forms of DIIHA, either for IgG, C3, or both. Rare cases with negative DAT reported either low antibody density, massive intravascular hemolysis, or red cell transfusion administered prior to testing [1,10]. When approaching a case where there is evidence of hemolysis and an established temporal relationship with a drug known to induce autoantibody production, investigation for drug-dependent antibodies can be performed in adequate laboratory settings, with quality ensured expertise and appropriate controls [1,3].

As there are no defining clinical features and drug-induced autoantibodies are indistinguishable from idiopathic autoantibodies, misdiagnosis in DIIHA is extremely common, with some patients dying because of late diagnosis. Furthermore, hemolysis and serological results can persist for up to months after withdrawal of the drug, leading to erroneous attribution of recovery on the administered treatment and not to the interruption of the drug [6].

If DIIHA is suspected, relevant medication should be withdrawn immediately. In mild cases, recovery usually happens within one to two weeks after drug suspension, with no other necessary measures. As most drugs have a relatively short half-life, even a strong drug-dependent antibody loses its effect shortly after drug suspension [1]. Failure to recognize DIIHA can have disastrous consequences, especially when patients present complaints for which the offending drug was prescribed. Regarding diclofenac DIIHA, some cases have been reported of patients receiving the drug again for “low back pain” during the early onset of hemolysis [9].

In acute severe DIIHA, recommendations suggest close monitoring of clinical and laboratory signs, early intravenous access, and fluid resuscitation. Blood transfusion should not be withheld in patients with severe anemia [3]. Even though crossmatch-compatible blood can be difficult to find, a review of 134 patients showed that 68 (55%) patients received blood transfusions. Though most patients (85%) received glucocorticoids, its benefit is uncertain and its routine use is not recommended as drug eviction is usually enough to stop the immune response [14].

Applying the criteria of the World Health Organization causality assessment method to assess a possible drug‐induced etiology of IHA we are confident the most likely diagnosis for our patient was diclofenac-induced IHA [17]. Our analysis is supported by: (a) the presence of autoantibodies in the patient’s eluate was demonstrated; (b) diclofenac is known to induce DIIHA; (c) there is a chronological relationship between hemolysis and diclofenac administration; (d) a review of the literature failed to show cases of DIIHA induced by any of the other drugs taken by the patient; (e) symptoms and laboratory signs significantly improved after discontinuation of diclofenac; (f) no coexistent neoplastic, autoimmune, or infectious diseases known to be secondary causes of AIHA were found after systematic and comprehensive studies; and (g) though glucocorticoids were started on admission, no recurrence of anemia was verified in a three-year follow-up period, which would be uncommon if the patient suffered from WAIHA.

## Conclusions

As a potentially fatal complication of a widely used drug in clinical practice such as diclofenac, early and correct diagnosis of DIIHA and prompt removal of the offending drug is crucial for the patient’s recovery. Our case highlights the need for both prescribing physicians and patients to be aware of this frequently underreported and usually misdiagnosed entity.

## Appendices

**Table 2:** Causality categories. Adapted from the WHO-UMC system for standardized case causality assessment. The usual approach is to choose one of the causality terms’ categories and to test if the various criteria fit the content of the case report. All assessment criteria should be reasonably complied to assume a category. The WHO-UMC causality assessment system can be used for the assessment of case reports of adverse drug reactions or drug-drug interactions. WHO-UMC, World Health Organization-Uppsala Monitoring Centre

Causality term	Assessment criteria
Certain	• Event or laboratory test abnormality, with plausible time relationship to drug intake • Cannot be explained by disease or other drugs • Response to withdrawal plausible (pharmacologically, pathologically) • Event definitive pharmacologically or phenomenologically (i.e., an objective and specific medical disorder or a recognized pharmacological phenomenon) • Rechallenge satisfactory, if necessary
Probable/Likely	• Event or laboratory test abnormality, with reasonable time relationship to drug intake • Unlikely to be attributed to disease or other drugs • Response to withdrawal clinically reasonable • Rechallenge not required
Possible	• Event or laboratory test abnormality, with reasonable time relationship to drug intake • Could also be explained by disease or other drugs • Information on drug withdrawal may be lacking or unclear
Unlikely	• Event or laboratory test abnormality, with a time to drug intake that makes a relationship improbable (but not impossible) • Disease or other drugs provide plausible explanations
Conditional/Unclassified	• Event or laboratory test abnormality • More data for proper assessment needed • Additional data under examination
Unassessable/Unclassifiable	• Report suggesting an adverse reaction • Cannot be judged because information is insufficient or contradictory • Data cannot be supplemented or verified
